# Neutrophil-to-lymphocyte ratio and mean platelet volume can be useful markers to predict sepsis in children

**DOI:** 10.12669/pjms.344.14547

**Published:** 2018

**Authors:** Adem Dursun, Serkan Ozsoylu, Basak Nur Akyildiz

**Affiliations:** 1 Adem Dursun, MD. Department of Paediatrics, Division of Intensive Care, Erciyes University, Faculty of Medicine, Kayseri, Turkey; 2 Serkan Ozsoylu, MD. Department of Paediatrics, Division of Intensive Care, Erciyes University, Faculty of Medicine, Kayseri, Turkey; 3 Basak Nur Akyildiz, MD. Associate Professor of Paediatrics, Department of Paediatrics, Division of Intensive Care, Erciyes University, Faculty of Medicine, Kayseri, Turkey

**Keywords:** Child, Neutrophil-to-lymphocyte ratio, Mean platelet volume, Sepsis

## Abstract

**Objectives:**

Neutrophil-to-Lymphocyte Ratio (NLR) and Mean Platelet Volume (MPV) have been found to be useful indexes for the diagnosis of sepsis in adults. However, the knowledge of their roles and cut-off values in pediatric patients is limited. The primary objective of this study was to assess the ability of NLR and MPV to predict sepsis in children. A secondary aim was to evaluate the comparison of these parameters with C-reactive Protein (CRP).

**Methods:**

The study was conducted on pediatric patients, who had two or more of the following criteria were included in the study: tachycardia, tachypnea, temperature change, leukocytosis, or leukopenia for age. Patients were classified into sepsis and non-sepsis groups. The sepsis group was defined as the presence of two or more age specific Systemic Inflammatory Response Syndrome (SIRS) criteria and increased Procalcitonin (PCT) level (>0.5 ng/ml)

**Result:**

The median age of the study population was 18 (6-169) months. Two hundred-sixty four episodes of sepsis were recorded in 125 patients. Eighty two were classified as sepsis and 182 as non-sepsis. CRP level and MPV value were significantly higher in the sepsis group compared to non-sepsis group. The median CRP level was 47.8 mg/dl (10.2-119.5) in the sepsis group and 18.6 mg/dl (4.9-66.1) in the non-sepsis group (p=0.006). In the sepsis group, the median MPV value was 8.4 (7.6-9.5) and it was 7.8 (7.1-8.5) in the non-sepsis group (p=0.001). Significant correlations were found between the procalcitonin (PCT) and CRP level (p<0.001; r = 0.279), NLR (p=0.02; r = 0.186) and MPV (p<0.001; r = 0.243). MPV had the highest specificity for predicting sepsis (75.8%). The largest AUC was 0.629 with a cut-off value 8.5 for MPV, while the AUC was 0.557 with a cut-off value 1.97 for NLR and 0.606 with a cut-off value 38.9 for CRP.

**Conclusions:**

NLR and MPV values should alert clinicians to the possibility of sepsis and to initiate or change antibiotic treatment.

## INTRODUCTION

Despite advances in modern antibiotics and intensive care practices, sepsis remains one of the leading causes of morbidity and mortality in adults and pediatric patients worldwide. According to recent estimates by the U.S. Centers for Disease Control, the rate of hospitalization for sepsis more than doubled between 2000 and 2008 (from 11.6 to 24 per 100,000 population) and now accounts for over 720,000 annual hospitalizations and $14.6 billion in healthcare expenditures annually in the United States.^1^

Following the 2001 consensus statement of the Society of Critical Care Medicine, Paediatric sepsis was defined the systemic inflammatory response syndrome in the presence of suspected or proven infection. Infections can be proven by positive culture, tissue stain, or polymerase chain reaction test.^2^ Early diagnosis of this condition can lead to significant improvements in the prognosis of sepsis. However, sepsis and non infectious Systemic Inflammatory Response Syndrome (SIRS) frequently produce very similar clinical features, particularly in children. Numerous biomarkers have been evaluated to distinguish sepsis from non infectious causes of SIRS, although none have been entirely useful. C-reactive Protein (CRP) and Procalcitonin (PCT) are currently the most frequently used biomarkers in clinical practice. PCT is considered to have a higher capacity to diagnose sepsis than CRP.^3^ However, these tests have limitations in terms of their high costs and time requirement, which place them practically out of the reach of developing countries. Therefore, clinicians still need to develop tools which are highly specific, highly sensitive, inexpensive, rapid and easy to measure.

The neutrophils and the lymphocytes are the key cellular component of the human host defense system against an infection. During the course of sepsis, the number of white blood cells may vary, depending on the stage of sepsis, the patient’s immunologic status, and the etiology of the infection.^4^ Increased number of neutrophils and decreased number of lymphocytes alert the clinician for an infection. Neutrophil-to-Lymphocyte Ratio (NLR) and Mean Platelet Volume (MPV) are simple biomarkers of inflammation which can be measured in routine hematological examination^5^,^6^ Although NLR and MPV have been found to be a useful index for the diagnosis of sepsis and many diseases in adult patients, their roles and cut-off values in pediatric patients with sepsis remain unclear.^7^,^8^

The aim of the present study was to investigate the ability of NLR and MPV in differentiating sepsis from non infectious causes of SIRS and to compare them with the traditional parameters CRP and PCT. To the best of our knowledge, these parameters have not been previously studied in children with sepsis.

## METHODS

A retrospective cohort study was conducted of pediatric patients at Erciyes University Pediatric Intensive Care Unit (PICU) in Turkey, from January 2015 to January 2017. Individual patient consent was not obtained since all data used in this study were acquired retrospectively from the laboratory information system without any additional blood sampling. The study was approved by the local ethics committee (with a reference number of 2017/448).

Two Pediatric Intensive Care fellows determined that the patients had clinical sings of sepsis at any point during hospitalization. Past medical history and the current clinical status combined to physical examination findings, laboratory tests and radiological imaging studies for each patient according to the clinical suspicion were assessed by the fellows. Those patients in the presence of suspected or proven infection and who had two or more of the following criteria were included in the study: tachycardia, tachypnea, temperature change, leukocytosis, or leukopenia for age. Patients with hematological disease, receiving chemotherapy and glucocorticoids were excluded.

### Data collection and definitions

Patient information regarding age, gender, clinical history and vital signs (body temperature, heart rate, respiratory rate, systolic and diastolic arterial pressure) was examined. If the patient’s body temperature exceeded 38.3 C or if the patient had clinical signs of sepsis, blood samples were obtained by venipuncture for the study of complete blood count, CRP and PCT on the same day. The MPV values were automatically calculated by devices in routine hemogram parameters. NLR values were obtained by dividing the neutrophil count by the lymphocyte count.

Patients were classified into sepsis and non-sepsis groups. The sepsis group was defined as the presence of two or more age specific SIRS criteria (Table-I), and increased PCT level (>0.5 ng/ml).^9^

**Table-I:** Pediatric SIRS criteria.

	Core Temperature (C)		Leukocyte count (leukocytes × 10^3^mm^3^)		Heart rate (beats/min)		Respiratory rate (breath/min)	SBP (mmHg)

Age group	Hypotermia	Hyperthermia	Leukopenia	Leukocytosis	Bradycardia	Tachycarda		
1 month to 1 year	<36	>38.5	<6	>17.5	<90	>180	>34	<75
2-5 years	<36	>38.5	<6	>15.5	NA	>140	>22	<74
6-12 years	<36	>38.5	<4.5	>13.5	NA	>130	>18	<83
13 to <18 years	<36	>38.5	<4.5	>11	NA	>110	14	<90

Adopted from (2), SBP: systolic blood pressure; SIRS: systemic inflammatory response syndrome; NA: not applicable

### Statistical analyses

Statistical analysis was performed using SPSS version 22.0 (IBM, Armonk, NY). The normality of parametric data was analyzed by the Shapiro Wilk test. Numerical variables were expressed as mean ± SD or median. Comparisons between groups for data with a normal distribution were performed using Student’s t-test, and the comparisons between groups for data that did not show a normal distribution were performed using the Mann-Whitney U test. Categorical variables were compared using the X^2^ test. The bivariate correlation tests were used to analyze the correlations. Whether NLR, CRP and MPV value were significant marker that differentiated groups with sepsis and non-sepsis was explored using 95% confidence intervals and area under ROC curve. When a significant area under the curve was obtained, the maximum possible sum of the sensitivity and specificity levels was considered the best cut-off point. Then, the sensitivity, specificity, positive and negative predictive values of the best NIRS cut-off points were calculated. A p value less than 0.05 was considered statistically significant.

## RESULTS

One hundred and twenty five patients who met the study criteria were included in this study. Some patients had multiple admissions to the PICU and 264 potential sepsis episodes of 125 patients were recorded for almost a two-year period. The median age of the study population was 18 (6-169) months. There were 63 (50.4%) males and 62 (49.6%) females.

Two hundred-sixty four data were classified as sepsis (n=82) and non-sepsis (n=182). The median CRP level was 47.8 mg/dl (10.2-119.5) in the sepsis group and 18.6 mg/dl (4.9-66.1) in the non-sepsis group. When the median MPV values compared between two groups it was 8.4 (7.6-9.5) in the sepsis group and 7.8 (7.1-8.5) in the non-sepsis group. CRP level and MPV value were significantly higher in the sepsis group compared with the non-sepsis group (p=0.006, p= 0.001 respectively). When the mean NLR levels were compared between the two groups it was 3.4 (1.9-6.1) in the sepsis group and 2.9 (1.2-6.0) in the non-sepsis group (p = 0.141).

Significant correlations were found between the PCT and CRP level (p<0.001; r = 0.279), NLR(p=0.02; r = 0.186) and MPV (p<0.001; r = 0.243). A statistically significant relationship was found with a positive correlation between CRP and MPV(p=0.01; r =0.159) and NLR (p<0.01; r=0.229). No correlation was determined between MPV and NLR (p=0.776).

**Fig.1:**
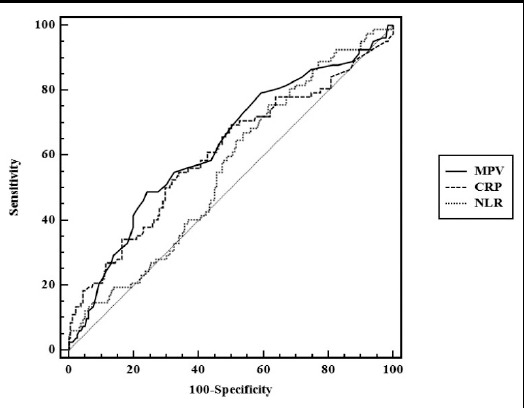
Comparison of ROC curves of MPV, CRP and NLR.

The ROC curves of the three parameters differentiating sepsis from non-sepsis are presented in Fig.1. The sensitivity, specificity, PPV and NPV of CRP, NLR and MPV were calculated on the basis of the ROC curves (Table-II). MPV had the highest specificity for predicting sepsis. Optimal cut-off values and AUCs of the parameters are shown in Table-III. The largest AUC was 0.629 for MPV, while the AUC was 0.557 for NLR and 0.606 for CRP.

**Table-II:** Sensitivity, Specificity, PPV and NPV of markers.

Markers	Sensitivity (%)	Specificity (%)	PPV (%)	NPV (%)
CRP	54.8	65.9	42.1	76.4
NLR	75.6	38.4	35.6	77.8
MPV	48.7	75.8	47.6	76.7

PPV: Positive Predictive Value; NPV: Negative Predictive Value; CRP: C-reactive protein (mg/l); NLR: Neutrophil-To-Lymphocyte Ratio; MPV: Mean Platelet Volume.

**Table-III:** Area under the ROC curves, cut-off values and P-values of each tested markers.

Markers	Cut-off	AUC	P-value	% 95 CI
CRP	38.9	0.606	<0,001	0.544-0.665
NLR	1.97	0.557	0.143	0.495-0.618
MPV	8.5	0.629	0.007	0.568-0.688

AUC: Area Under The Receiver Operating Characteristic Curve; CI: Confidence Interval; CRP: C-reactive protein (mg/l);NLR: Neutrophil-To-Lymphocyte Count Ratio; MPV: Mean Platelet Volume.

## DISCUSSION

The present study is perhaps the first to investigate MPV independently to predict bacterial sepsis in pediatric patients. We also showed the correlation between PCT and CRP, NLR, and MPV values.

Early diagnosis of sepsis in PICU improves the outcome of the patients, but there are nonspecific symptoms and the diagnosis is difficult, particularly in children.^10^ Therefore, specific markers and molecular diagnostic assays are still needed in order to improve its clinical management. Several studies have investigated whether PCT and CRP could be useful biomarkers.^8^ However, they take a long time for the results to be attained (>24 hours for PCT, > 6 hours for CRP in our hospital) and these methods remain expensive in developing countries such as Turkey. In the present study, PCT was considered as a reference control and raised PCT levels were correlated with CRP, NLR, and MPV. CRP had a better correlation with PCT than did NLR and MPV. Moreover, our results also identified that increases in CRP values were correlated with NLR and MPV. A study of adults with suspected bacteremia and sepsis, which grouped the patients according to PCT levels, determined correlations between PCT and WBC, CRP and NLR. The highest correlation value was found between PCT and NLR.^11^ According to our findings, CRP showed the strongest correlation coefficient among all of the evaluated parameters. There may be indications for measuring PCT and CRP in acute medical patients, such as, for monitoring the antimicrobial treatment and progression of illness. Positive correlations of PCT with CRP, MPV and NLR showed that monitoring of MPV and NLR may play a role in the follow-up of septic pediatric patients. When elevated MPV and NLR levels return to normal, this may indicate either the disappearance of sepsis or an improvement in the patient’s condition.

MPV is easily calculated and immediately available from the complete blood count. Several studies have demonstrated that MPV increases in septic patients and it has been hypothesized that activated platelets are altered in terms of shapes and sizes.^12^ Larger platelets are functionally, metabolically, and enzymatically more active than smaller ones and produce chemokines and cytokines.^13^ In the literature many studies have discussed the changes in MPV values in septic patients specifically in adults and neonates. Catal et al. grouped the preterm infants in their study as control (n=100) and sepsis (n=91). They showed a significant increase in the MPV in the case of the sepsis group and reported that MPV may be a useful predictor in diagnosis, a value of 10.35 fl was identified as the cutoff, probably resulting in sepsis with a sensitivity of 97.8% and specificity of 78.7% (AUC = 0.949; p < 0.001).^14^ In adults, Ates et al. determined higher MPV values in septic patients than in the control group and MPV was found to be have a cut-off 8.85 fl, sensitivity of 69.6%, and specificity of 62.5%.^15^ In a cohort study of 43 pediatric patients with acute pyelonephritis and the authors investigated higher MPV and the cut-off value was 8.2 fl(AUC: 0.906), with a sensitivity of 81.4% and specificity of 86.3%.^16^ In concordance with the previous study, our results found that MPV values were higher in patients with sepsis compared to those without sepsis. At a cut-off value of 8.5 fl, the sensitivity of the MPV was 48.7%, and the specificity was 75.8%. The present study found that MPV had a superior discriminative value to that of CRP (AUC: 0.629 and, 0.606 respectively) and MPV performed a better performance than CRP in differentiating true bacterial sepsis (47.6% and 42.1%, respectively).

As the physiological immune response to bacterial infection, changes in blood cell components are often characterized by an increase in neutrophils and a decrease in lymphocytes.^17^ NLR has been proposed for use as an additional infection marker and it is a potentially interesting parameter in predicting bacteremia.^17^ Different NLR cut-off values have been published for the diagnosis of sepsis. However, the ideal cut-off is controversial. In an adult study an NLR > 5 was suggested to be a more convenient marker than CRP.^11^ In a retrospective study in children involving patients with bacterial pneumonia and viral pneumonia, the NLR cut-off value for bacterial pneumonia diagnosis was found to be 1.7 with a sensitivity of 74.2% and specificity of 76.2%.^18^ These findings are different to that demonstrated by the present study. We identified that an optimal cut-off value of 1.97 resulted in 75.6% sensitivity and 38.4% specificity. We demonstrated that NLR is not a good marker in differentiating sepsis from non sepsis in pediatric patients. Our study showed that a higher NLR was found in patients with sepsis when compared to patients without sepsis. However, the diagnostic performances of MPV and CRP were found to be superior to that of NLR. The AUCs were 0.629, 0.606 and 0.557, respectively.

The ability of NLR and MPV to predict sepsis in pediatric patients has not been studied before. The present study showed NLR and MPV may be a helpful clinical tool for use in PICU and pediatric emergency departments, when considering that PCT and CRP plasma concentration measurement remains expensive and a long time is required for the results to be attained.

### Limitations of the study

We undertook a single center observational study and used patient records, data on previous antibiotic use were not available. Additionally, the small sample size is a limitation of this study. A larger sample size and further investigation to determine the effect of these parameters are needed.

## CONCLUSION

NLR and MPV values were higher and correlated with PCT and CRP in pediatric patients with sepsis. It should be noted that the importance of PCT and CRP remain unchanged and ever, NLR and MPV values should alert clinicians to the possibility of sepsis and to initiate or change antibiotic treatment.

### Authors’ Contribution

**BNA and SO** did data collection and manuscript writing.

**AD** did statistical analysis and editing of manuscript.

**AD** takes the responsibility and is an accountable for all aspects of the work ensuring that questions related to the accuracy or integrity of any part of the work are appropriately investigated and resolved.
